# Why Does Cognitive Reserve Align With Modern Western Ideals of Success? Theoretical & Methodological Proposals

**DOI:** 10.1093/geroni/igab046.2640

**Published:** 2021-12-17

**Authors:** Douglas Hanes

**Affiliations:** Stony Brook University, Stony Brook, New York, United States

## Abstract

Cognitive reserve (CR) is a framework that investigates discrepancies between brain pathology and cognitive decline. In explaining why individuals with similar levels of brain pathology display different levels of functional impairment, CR research focuses on factors that resemble modern, Western ideals of success: greater education, professional achievement, a self-directed life, and physically and intellectually stimulating leisure time. This theoretical paper documents this alignment between CR and modern, Western ideals of success to hypothesize different mechanisms by which CR may operate. The focus in the CR literature has been on investigating and operationalizing the direct cognitive changes that come from intellectual cultivation, and the native abilities that are hypothesized to produce differences in both education and cognitive outcomes. This paper argues that an attention to CR’s relationship to current definitions of success presents alternative hypotheses about the mechanisms by which CR operates. Specifically, the paper outlines two potential mechanisms and frames alternative means of studying them: First, does the accrual of CR simply follow from being successful in conventional ways because of the material benefits of wealth and stability that success brings? Second, does a lack of success carry cognitive risks solely because of material deprivation, or are there additional psychosocial penalties that come from living a non-normative life—especially when that is not of one’s choosing? This paper proposes both cross-cultural and intersectional methods to begin to better understand the relationship between normative success and cognitive health.

